# Predation by avian insectivores on caterpillars is linked to leaf damage on oak (*Quercus robur*)

**DOI:** 10.1007/s00442-018-4234-z

**Published:** 2018-08-16

**Authors:** Bengt Gunnarsson, Jonas Wallin, Jenny Klingberg

**Affiliations:** 1 Department of Biological and Environmental Sciences, University of Gothenburg, Box 461, 405 30 Göteborg, Sweden; 2 Department of Statistics, Lund University, Lund, Sweden; 3 Gothenburg Botanical Garden, Gothenburg Global Biodiversity Centre, Göteborg, Sweden

**Keywords:** Artificial caterpillars, Bird predation, Experimental manipulation, Canopy damage, Survival probability

## Abstract

Birds that are foraging in tree canopies can cause a substantial decrease in arthropod numbers. Trees may benefit from avian insectivores attacking insect herbivores. In a field study, we tested whether the intensity of bird predation on caterpillars is linked quantitatively to leaf damage caused by insect herbivores, a hypothesized relationship that previously was poorly investigated. Artificial caterpillars were placed in the lower part of oak trees (*Quercus robur*) in urban and suburban sites across the city of Gothenburg, Sweden. Two days later, we recorded the survival: the pooled predation rate was 11.5% (5.7% day^−1^). Mean predation rate per tree was 10.4%. Mean leaf damage, i.e. leaf area eaten by insect herbivores, per tree was 5.7% but there was large variation between trees. We found a significant negative relationship between survival probability of caterpillars and leaf damage in an analysis using a mixed model logistic regression. This suggests that caterpillars are at high risk of bird attacks in trees with a high degree of leaf damage and avian insectivores may increase the foraging effort in the foliage of such oak trees. Our findings concerning the quantitative relationship between the predator–prey interactions and plant damage suggested tentatively that the survival probability of caterpillars decreases rapidly at 15–20% leaf damage in lower part of oak canopies. Furthermore, our findings add credence to the idea of using artificial caterpillars as a means to obtain standardized comparisons of predation rates in various habitats.

**Electronic supplementary material:**

The online version of this article (10.1007/s00442-018-4234-z) contains supplementary material, which is available to authorized users.

## Introduction

Predation is one of the strongest interactions between individuals in ecological communities. The predator increases its fitness, while the prey dies and its fitness is zero, unless successful reproduction has been previously accomplished. Such interactions can be summed up in the prey and predator populations and further be translated into top-down cascading effects in food webs that ultimately can affect the primary producers, i.e. the green plants. But the intensity of predator–prey interactions varies between ecosystems. For instance, recent experimental data suggest that predation on insect herbivores, in terrestrial food webs, is more common in the tropics, close to the equator, and at lower elevations than close the poles and at higher elevations (Sam et al. 2015; Roslin et al. 2017). The prevalence of high predation pressure in the tropics has long been advocated by researchers on theoretical grounds (e.g. MacArthur 1969; Schemske et al. 2009). However, predation seems to play a significant role in many types of terrestrial systems, including those at higher latitudes (e.g. Abrams 2000; Gilg et al. 2003; Jost et al. 2005).

Recent reviews and meta-analyses show that birds and other insectivorous vertebrates have a significant impact on lower trophic levels in terrestrial food webs (e.g. Gunnarsson 2007; Van Bael et al. 2008; Mooney et al. 2010; Mäntylä et al. 2011). Such insectivores reduce the negative effects on the primary producers that are caused by, e.g. insect herbivores. This important “ecosystem service” by avian insectivores has been recognized during the latest decades and the empirical evidence is now plentiful (Whelan et al. 2016). The positive effect of bird predation on plants has been recorded in multiple ecosystems and habitats, at different latitudes and in various climates. There are, however, several gaps in the knowledge regarding the ecological mechanisms that affect the relationship between insectivorous birds and their arthropod prey. Examples of such information gaps include the importance of species richness of birds for depressing arthropod densities in agroecosystems (Philpott et al. 2009); the interaction between the quality of plants for insect herbivores and bird predation effects (Singer et al. 2012); the role of conditional trophic cascades in systems driven by bottom-up forces that can switch to top-down (bird predation) due to water availability (Bridgeland et al. 2010); and the possibility of more effective predation by birds with increasing plant species diversity (Muiruri et al. 2016). Another gap of knowledge concerns the exact relationship between the predation rate on insects by avian insectivores and plant damage. Such data would be helpful to researchers who want to predict the contribution of bird predation to the ecosystem service of pest regulation (Whelan et al. 2016).

Here we examine damages on leaves of English oak (*Quercus robur*) in relation to predation pressure by birds. To test the hypothesis of a connection between the probability of survival of insect herbivores and the consumption of leaf tissue by insects, we conducted a field study with experimental manipulation of insect prey. Artificial caterpillars were placed on the branches of oak and we recorded attacks on the larvae. We also measured leaf damage on the branches. In addition to the null hypothesis, there are two alternatives; either, there is a positive relationship between the probability of caterpillar survival and leaf damage, or there is a negative relationship. The first hypothesis suggests that the birds are avoiding foraging in canopies with highly damaged leaves. This could be the case if, e.g. risk of predation by avian predators (e.g. sparrowhawk *Accipiter nisus*; see Götmark and Post 1996) on foraging insectivorous birds is increasing in proximity to trees with high levels of leaf damage. The second hypothesis suggests that avian insectivores are increasing their foraging efforts in trees with high levels of damage, due to high population density of insect herbivores. In the light of earlier studies in other systems, e.g. tropical forests (Sam et al. 2015), we expect the second hypothesis to be supported by our study.

## Materials and methods

### Study sites

Field experiments were conducted at eight urban and suburban sites across the city of Gothenburg, SW Sweden (population ca 540,000) in June 2016. All sites were part of mixed woodland, or park, with English oak as a predominant species (Table 1). Canopy cover of the experimental areas differed, as well as the density and species composition of the understory between the sites, partly due to variation in the management. At some of the sites, there was no active management except for maintenance of paths or walkways (3 Skatås, 4 Delsjöområdet, 5 Guldheden). At other sites, there was occasional maintenance such as removal of bushes and small trees, especially close to walkways (1 Ramberget, 2 Sörhallsparken, 6 Slottsskogen, 8 Botaniska trädgården). At one site (7 Skansen Kronan), there was maintenance of lawns that were regularly mown between large veteran trees but in some parts of the area, bushes and graminoids were left uncontrolled. Conceivable effects of variation in management at our sites were assessed by vegetation measurements (canopy cover and leaf area index) and monitoring of avian insectivore abundance (Table 1).

**Table 1:** Experimental sites in Gothenburg: type of habitat and experimental details (number of trees/caterpillars/leaves)

Site	Habitat	Number of trees/caterpillars/leaves	Canopy cover (%)	*L*_e_	No. individuals (mean ± SD)
1. Ramberget	Urban park	4/64/100	80.3	2.5	12.0 ± 2.6
2. Sörhallsparken	Urban park	5/59/90	33.1	0.9	17.3 ± 2.5
3. Skatås	Suburban woodland	5/64/100	No data	No data	14.0 ± 5.0
4. Delsjöområdet	Suburban woodland	6/62/100	98.7	4.1	14.7 ± 4.7
5. Guldheden	Urban woodland	4/66/100	100.0	3.9	17.0 ± 3.0
6. Slottskogen	Urban park and woodland	4/64/100	96.2	3.6	16.0 ± 3.6
7. Skansen Kronan	Urban park	4/65/100	42.7	2.0	5.3 ± 2.1
8. Botaniska trädgården	Urban woodland adjacent to botanical garden	4/64/100	95.5	4.6	20.3 ± 4.2

Canopy cover and effective leaf area index (*L*_e_) based on LiDAR data. Number of individuals of insectivorous birds recorded at each site

At each site, the experiment was carried out within an area of approximately 1/2–1 ha. A central circular plot with a 10 m radius (area 314 m^2^) was chosen for estimation of canopy cover (%) and effective leaf area index (*L*_e_). Within each circular plot, canopy cover was estimated based on a canopy digital surface model in 1 m resolution derived from aerial LiDAR (light detection and ranging) data. The Gothenburg LiDAR data were obtained in October 2010. Further details of the data and data processing can be found in Klingberg et al. (2017). The methodology was developed for mapping effective leaf area index (*L*_e_), which revealed a high positive correlation to canopy cover (Klingberg et al. 2017). One of the experimental sites, 3 Skatås, was not covered by the Gothenburg LiDAR data. In our analyses, we used data from a nearby site (4 Delsjöområdet, ca 2 km distance from site 3) with similar environmental conditions (visual assessment) and abundance of avian insectivores (Table 1) as replacement for missing data.

Prior to the start of the experiment, birds were monitored by point counts. At each site, all birds that were seen or heard during 5 min were counted at a central point in our experimental area (i.e. not restricted to circular plot). This was done three times (June 7th, 9th, and 13th) on days without rain or strong wind. The sequence of the sites varied on each sampling occasion but the time for monitoring the birds was the same, i.e. between 04.00 am and 08.30 am local time. We regarded insectivores to be relevant for this study and the mean number of individuals at each site is shown in Table 1. Some of the most common species were, e.g. blue tit (*Cyanistes caeruleus*), great tit (*Parus major*), willow warbler (*Phylloscopus trochilus*), and chaffinch (*Fringilla coelebs*). Both adults and juvenile birds were counted. Previous studies in similar habitats in SW Sweden have shown that these species, together with other insectivores, can significantly reduce abundance of canopy-living arthropods (Gunnarsson et al. 2009; Heyman and Gunnarsson 2011). Multiple studies in European forests and orchards have revealed that both blue tit and great tit diligently attack caterpillars on twigs and leaves in deciduous trees (e.g. Naef-Daenzer and Keller 1999; Naef-Daenzer et al. 2000; Mols and Visser 2002). Blue tit seems to prefer tree-tops and the outermost twigs, whereas great tit and chaffinch are evenly dispersed in vertical strata of deciduous canopies (Böhm and Kalko 2009).

### Experimental methods

**Fig. 1:**
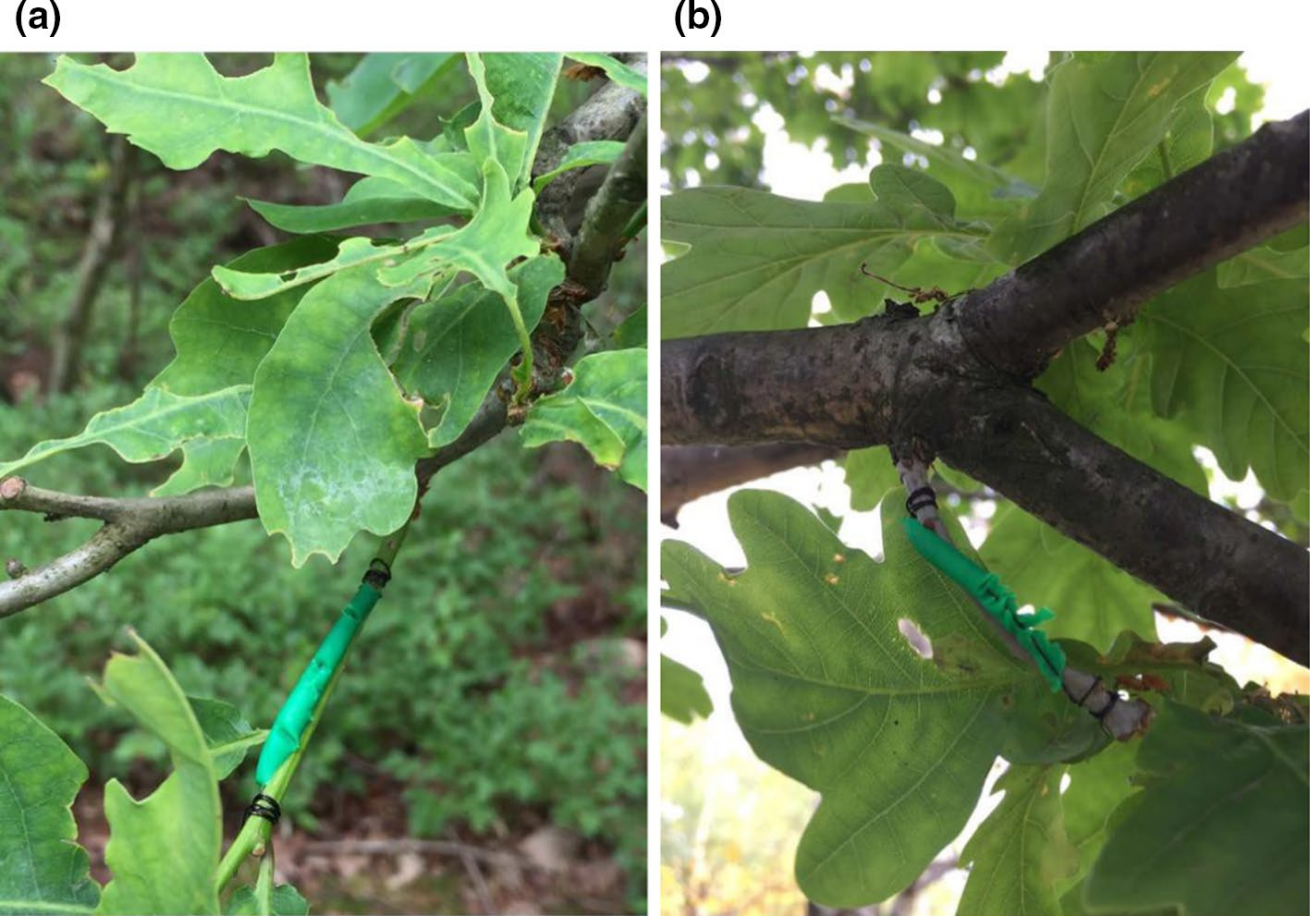
Bird predation on artificial caterpillars. **a** Beak and **b** hack marks on plasticine larvae on oak branches. Colour version is available online. Photo: Sara Elg (colour figure online)

We used artificial caterpillars to assess the predation rate by birds. Each larva was made of light green plasticine (non-toxic clay: “Giotto Patplume”, colour was “verde chiaro” i.e. light green) and attached to a thin black metal wire (*Ø* 0.3 mm). The hand-made larvae had a mean length of 26.3 mm (s.d. 2.7, *n* = 30) and a mean diameter of 2.5 mm (s.d. 0.2). Thereby, they were reasonably similar to various caterpillars in the wild, e.g. the winter moth (*Operophtera brumata*) and autumnal moth (*Epirrita autumnata*) which are common species throughout Sweden (Elmquist et al. 2011). The artificial larvae were placed on branches (1–2 m above the ground) of English oaks of various sizes at eight sites (Fig. 1). Tree numbers per site varied between 4 and 6 (Table 1). In total, 36 oak trees were included. To be selected, trees had to have branches with a well-developed green foliage that we could reach from the ground. We aimed at including various sizes of oak trees in the total sample. At each site, we installed between 59 and 66 caterpillars at the start of the experiment (Table 1). We secured 6–7 larvae on each branch, with a distance between caterpillars of at least 40 cm. The number of branches with attached caterpillars varied between 1 and 3 per tree, because of differences in the availability of branches.

The caterpillars were installed on 14 June 2016 at four sites and 15 June 2016 at the remaining four sites. The predation rates were recorded after approximately 48 h. The artificial larvae that had been attacked were considered to be eaten by birds. We observed mainly two types of attacks; first, caterpillars with single or multiple beak marks and second, caterpillars that had been “hacked”, i.e. vigorous hack marks that had caused considerable damage to the plasticine larvae (Fig. 1). Such attacks are caused by birds (Low et al. 2014). In addition, we observed artificial larvae where we could not with certainty identify what had caused the damage (in total 8.5%, see “Results” section). We took a conservative approach in estimating survival rate of caterpillars and considered those specimens that had clear beak or hack marks to be dead (see “Results” section). In our analyses of artificial caterpillars, we focussed on survival probability in each tree because they are entities separated from each other and can be viewed as biological individuals (in most cases). Birds usually move between them by flight, meaning that a behavioural “decision” that may involve changes in foraging, risk of predation, etc., has to be taken. Several other studies of interaction between avian insectivores and their arthropod prey in tree canopies have used tree individuals as study unit (e.g. Gunnarsson 1996; Mols and Visser 2002; Heyman and Gunnarsson 2011; Bereczki et al. 2014).

### Estimation of leaf damage

We randomly sampled oak leaves in the lower part foliage of our experimental trees. Ten leaves were collected from each branch with installed artificial caterpillars. The leaves from each branch were selected based on random numbers that were assigned each chosen leaf, and counting was starting from the branch tip. In total, we collected 790 leaves from 79 branches (Table 1). We restricted our sampling to the outer layer of foliage because any damages on those leaves will be visible to birds at a distance. The amount of damage on leaves was given by the mean percentage of leaf area missing. It was assumed that mainly caterpillars caused the damages. This assumption was supported by observation of caterpillars of moths (Geometridae) in our experimental sites. We used a mean value of leaf area missing for each tree based on measurements of 10, 20 or 30 leaves, depending on the number of experimental branches. The area missing on each leaf was measured using the software “Win Seedle v. 5.1a”. First, the leaf was scanned and the area was recorded in mm^2^. Second, the missing (consumed) areas were manually adjusted and the area of the intact leaf was recorded. Third, the area eaten was estimated as the difference between the first and second measurements. In our analyses, we used the percentage of leaf damage for the quantification of missing leaf surface but we also examined the relationship between the percentage of leaf damage and missing area in mm^2^ (see “Results” section). All measurements were done by one person. Our experimental trees were not selected based on leaf damage but on the availability of branches, i.e. if branches were possible to reach from the ground. Therefore, there was no expectation in advance of the amount of leaf damage in any of the trees.

### Statistical analyses

We used logistic regression to examine survival probability of artificial caterpillars in relation to leaf damage. We assumed that within each tree the probability of survival is the same for each caterpillar. To link the leaf damage to the survival probability, we used a logistic link function, i.e. the survival probability of a caterpillar at a specific tree k is

1 $$ p_{k } = g(\beta_{0} + x_{k}  \beta_{1} ) = \frac{{{\text{e}}^{{\beta_{0} + x_{k} \beta_{1} }} }}{{1 + {\text{e}}^{{\beta_{0} + x_{k} \beta_{1} }} }}. $$

Here *β*_0_ is the base effect and $x_{k}  \beta_{1}$ is the effect of the leaf damage, *x*_*k*_, on the survival probability, $p_{k }$, of a caterpillar in tree *k*. The function *g* is often denoted as the inverse-logit function. If a regular logistic regression model was fitted to the data this would assume that survival of caterpillars on different trees have the same survival probability, after removing the effect of leaf damage for each tree. This is a strong assumption, as there could have been other covariates influencing the survival probability of the caterpillars on a fixed tree, e.g. the number of nearby trees, the tree’s position in the park, or the soil contents near the tree, etc. Ignoring these covariates can give false significant effects (Fitzmaurice et al. 2012). Although the design of the experiment reduces the effect of this issue, we took the precaution of using a mixed model, where a random effect is added for each site to the survival probability, i.e.

2 $$ p_{k} = g(\beta_{0 } + x_{k } \beta_{1} + Z_{j(k)} ) , $$

where *Z*_*j*(*k*)_ is a normal random variable with zero mean and unknown variance, and *j(k)* is the index for the site of the *k’*th tree. The analysis was performed in R (R core team 2017) using the lme4 package (Bates et al. 2015). The code for reproducing the analysis is included in ESM Appendix 1.

We also used non-parametric correlation analysis for the relationship between two variables used for estimation of the leaf damage.

## Results

### Predation rate

In total, 508 artificial caterpillars were installed and exposed to predation by avian insectivores. However, there were uncertainties regarding predation on 43 artificial larvae (8.5%, i.e. 43/508). In 32 caterpillars, we were unable to discern whether the larvae had been damaged by bird predators or by other causes (e.g. other predators, whipping branches, or fallen off the wire). In our further analyses, these caterpillars were considered to be “alive” at inspection. We consider this a conservative estimate because in addition to the 32 caterpillars with unknown cause of injuries there were another 11 larvae where we suspected attacks from bird but the marks on the larvae were not conclusive evidence. These 11 caterpillars were excluded from predation estimates. This means that 497 artificial larvae were left for analysis.

**Fig. 2:**
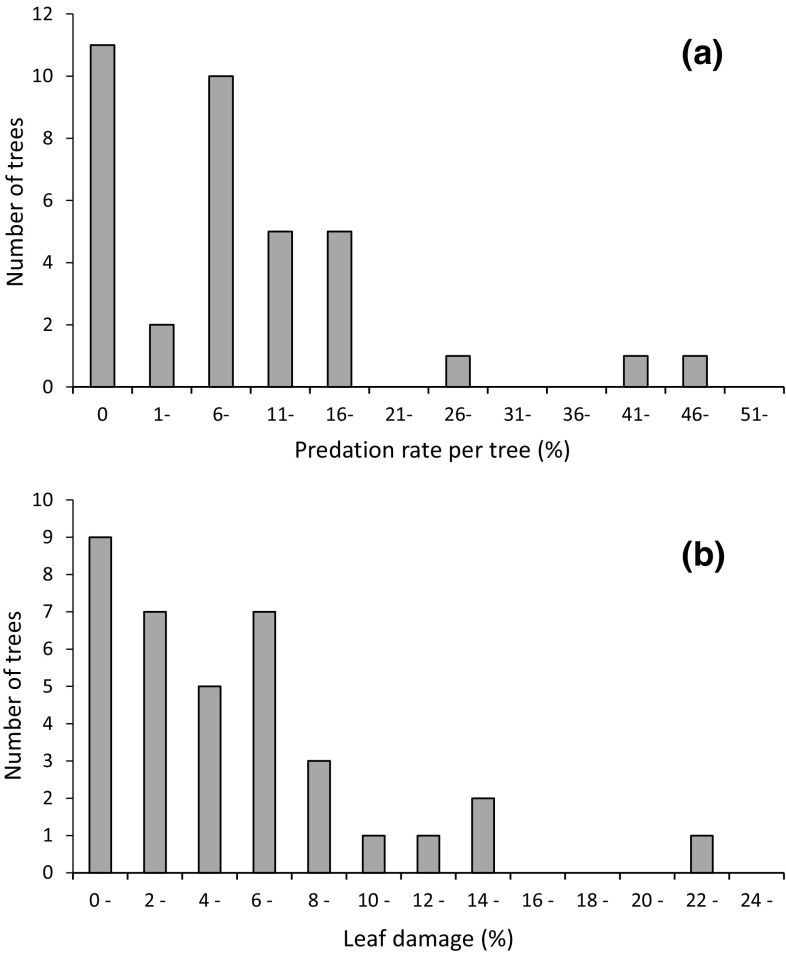
Frequency distribution of **a** predation rate on artificial caterpillars and **b** leaf damage per oak tree (*n* = 36). A mean percentage of leaf damage is shown for each tree

After 2 days, predation was recorded for 57 caterpillars on which beak or hack marks were observed. Predation rate was 11.5% (57/497), i.e. 5.7% day^−1^, for all caterpillars pooled. The number of caterpillars installed in each tree varied between 6 and 20, and mean number of attacked caterpillars per tree was 1.6 (s.d. 2.0). The mean predation rate per tree was 10.4% (s.d. 11.0%). In 31% of the experimental trees, there was no attack recorded (Fig. 2a). But in the majority of trees (61%), predation rate varied between 5 and 18% (Fig. 2a). Among the attacked caterpillars, 37% (*n* = 21) exhibited single or multiple (up to six) beak marks (Fig. 1a), whereas 63% (*n* = 36) showed hack marks (Fig. 1b), i.e. marks caused by a more or less constant pecking on the caterpillar body.

### Leaf damage

The mean leaf damage, i.e. loss of leaf area, per tree was 5.7% (s.d. 4.8%) based on mean values from each tree. But there was large variation in leaf damage between different trees. There was no tree without any damage: the lowest recorded damage per tree was 0.2% and the highest value was 22.2%. In 78% (*n* = 28) of the experimental trees the damage was below 8% (Fig. 2b). In our analyses, we express the leaf damage as the mean percentage (or proportion) of leaf area missing in each tree. However, there was a significant positive correlation between leaf damage (mean percentage per tree) and area missing (mean value per tree) estimated in mm^2^ per leaf (Spearman rank correlation *r*_s_ = 0.925, *P* < 0.001, *n* = 36). This means that a leaf damage measured as a high percentage of missing leaf area corresponds to a large area of missing leaf surface.

### Caterpillar survival in relation to leaf damage

We analysed the relationship between probability of caterpillar survival and estimates of leaf damage for each oak tree. In addition to the covariate leaf damage, we also measured canopy cover, effective leaf area index (*L*_e_) and abundance of birds. We omitted including the covariate “canopy cover” in the analysis given that the covariate had a strong correlation (correlation coefficient of 0.49) with the leaf damage, and thus we would not be able to separate the effect between leaf damage or “canopy cover”. We estimated the remaining models using Akaike information criteria (AIC). We only used the model that had leaf damage as it had the lowest AIC (Table 2). Although AIC is not ideal for mixed effect models, it was used here since the standard deviation (*σ*_sites_) of the random effect (site) basically did not change between models (Table 2), and therefore it was considered an adequate model selector in the present situation. Moreover, the small *σ* gives an indication that the spatial dependence is not very strong in our data (Table 2). The code for AIC comparison is included in ESM Appendix 2.

**Table 2:** Model estimates with Akaike information criteria (AIC) on survival of caterpillars with “leaf damage”, effective leaf area index “*L*_e_” and abundance of “birds”

Model	AIC	*σ*_sites_
Leaf damage + *L*_e_ + birds	124.6	0.2875
Leaf damage + birds	123.3	0.3043
Leaf damage + *L*_e_	122.7	0.2894
Leaf damage	121.6	0.3135

Standard deviation (*σ*) for study sites is shown

**Fig. 3:**
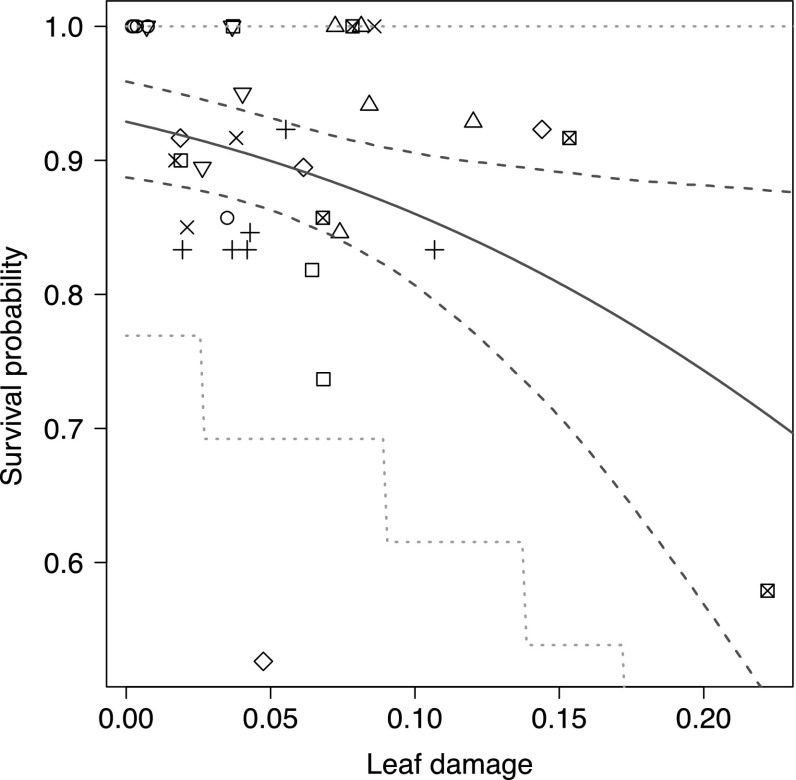
Survival probability of artificial caterpillars in relation to proportion of leaf damage in each tree (*n* = 36). The dashed lines indicate the 95% bootstrap confidence interval, the solid line is the maximum likelihood estimate. The dotted lines indicate the 95% predictive interval. Each point corresponds to the number of surviving caterpillars in a tree divided by the total number of caterpillars in the tree. The eight sites are denoted by different types of points

There was a significant negative relationship between leaf damage and survival of caterpillars recorded after 48 h exposure to predation by birds (Fig. 3). The 95% confidence bounds for the survival probability function (with “leaf damage” as argument) were estimated by the parametric bootstrap method (Efron and Tibshirani 1994). The same method was also used to generate the 95% predictive interval for a tree with thirteen caterpillars (median number) on it. The maximum likelihood estimate of the coefficient *β*_1_, the effect of leaf damage (in %) on survival probability, was − 7.5. The *P* value for the coefficient was 0.0075. Data used for the analysis are included in Electronic Supplemental Material (caterpillarTree.csv).

Consequently, our findings can be summarized as: avian insectivores foraging in the canopy of English oak had highest impact on caterpillars (i.e. lowest survival probability of caterpillars) in trees with the highest levels of leaf damage. The artificial caterpillars were exposed to the highest risk of predation by birds in oak trees with extensive leaf damage.

## Discussion

In this study, we show that the probability of survival of artificial caterpillars is significantly negatively affected by the proportion of damage on oak leaves. Our findings contribute to bridging a knowledge gap concerning the quantitative relationship between the predator–prey interactions and damage to plants. The probability of survival of (artificial) caterpillars, based on data from the field experiments, can be linked to measured leaf damage by means of logistic regression with random effects. The estimated relationship between caterpillar survival and leaf damage is shown in Fig. 3. Future studies could improve knowledge about variation in the relationship between the probability of survival of insect herbivores and damages in the foliage in other tree species or habitats. Such information can be useful for the interpretation of the efficiency of avian insectivores as agents of biological control in different environments (e.g. Mols and Visser 2002; Jedlicka et al. 2011; Karp et al. 2013; Maas et al. 2013).

A negative relationship between survival of caterpillars and leaf damages shows that bird predators are reducing insect herbivores in response to leaf damages. The negative relationship can be maximized during the caterpillar peak when consumption rate of leafs also is high. To maximize bird predation rate, caterpillars need to be abundant and above a certain size. For instance, studies on great tit and blue tit showed that the average searching time per prey item can be strongly reduced during caterpillar peaks (Naef-Daenzer and Keller 1999). Average searching time was low when birds were tending to return to the same foraging site in the canopy. This was caused by a clumped distribution of caterpillars. However, caterpillars needed to reach a certain size to be preferred over alternative prey such as spiders (Naef-Daenzer et al. 2000). This suggests that the abundance and size of caterpillars in foliage will greatly influence the survival probability. However, during years with mass occurrence of caterpillars the bird predators may have limited possibilities to control the prey populations, at least in Scandinavian forests at high latitudes (e.g. Hogstad 2005). This also have implications for the outcome of trophic cascades, i.e. indirect effects of predators that are spreading downward through food webs (Ripple et al. 2016). Studies on the indirect effects of bird predation on leaf damage of English oak in central Europe showed large local variations due to, e.g. tree age and forest composition (Böhm et al. 2011). Our approach in the present study can help in assessing the importance of bird predation in different sites. Reduction of leaf mass in deciduous trees by caterpillars´ consumption may elicit visual signals from trees to insect predators, e.g. avian insectivores (e.g. Koski et al. 2017). Consequently, the relationship between caterpillar survival probability and leaf damage can indicate whether birds are responding to “cry for help” from trees. A negative slope indicates that birds do respond to the signals from trees, whereas if the slope is zero this indicates that birds do not react to such signals.

The outcome of our experiment is congruent with the hypothesis that the bird predators increase their foraging efforts in trees that have leaves with high levels of damage. Birds are generally considered to be visually hunting predators (e.g. Holmes 1990). Studies of the insectivorous black-capped chickadee *Poecile atricapillus* showed that individuals can quickly learn to use leaf damage as a cue for finding prey (Heinrich and Collins 1983). There were also distinct differences in behaviour between morphologically closely related species of avian insectivores foraging in various types of foliage structure (Whelan 2001). Moreover, vegetation structure affected the bird predation pressure on arthropods living on branches of coniferous trees (Gunnarsson 1996). These examples of experimental studies show that insectivorous birds use their sight when foraging and that structural variation in the foliage guide them. Consequently, in the present study, it seems reasonable to suggest that damaged oak foliage with missing leaf areas is a strong cue for birds to locate abundant insect prey. But recent studies show that also volatile organic compounds from damaged trees can be used by birds to find foliage with high abundance of insect herbivores (e.g. Mäntylä et al. 2004, 2008; Amo et al. 2013). Avian insectivores are likely to use both visual and olfactory cues to identify tree individuals with high abundance of insects in the foliage, but the exact mechanisms are still largely unknown (e.g. Koski et al. 2015, 2017).

Our data suggest a large decrease in the survival probability of caterpillars when there is a 15–20% reduction of the leaf area (Fig. 3). If birds are using visual cues in combination with volatile organic compounds, such a decrease in survival probability might suggest that the signals from the trees are increasing rapidly within this interval of leaf damages. However, this tentative interpretation is based on probability of survival of caterpillars in a limited number of trees, which means that this suggestion needs to be confirmed by other studies.

There are multiple studies on the effects of bird predation on leaf-chewing insects in the foliage of various tree species, including oaks. Many of those investigations are experimental studies that focus on leaf damage, particularly missing leaf area, in relation to manipulated predation rate by avian insectivores on herbivorous insects (e.g. Marquis and Whelan 1994; Lichtenberg and Lichtenberg 2002; Barber and Marquis 2009; Bridgeland et al. 2010; Böhm et al. 2011). Typically, they use exclosures that make bird foraging on oak branches, or saplings, impossible and “control branches” with natural bird predation rates. A problem with the experimental design using exclosures is that the predation rate inside the exclosures decreases to zero, or close to zero. Moreover, the predation rate may vary greatly in the trees that are exposed to natural conditions. Thus, the expected variation in predation rate under natural conditions might be overlooked in these experiments. We, therefore, used artificial caterpillars as a means to estimate the effects of variation in predation rate. To our knowledge, the present study is the first experimental evidence of a quantified negative relationship between caterpillar survival and leaf damages in lower part of tree canopies.

Artificial caterpillars have been used in multiple studies to quantify predation pressure in testing a multitude of hypotheses (Lövei and Ferrante 2017). For instance, in a study of avian predation rate on artificial caterpillars together with experimental damages that were simulating insect herbivores in Papua New Guinea, Sam et al. (2015) showed that both birds and ants were attracted by mechanically damaged trees. This suggests that multiple predators can identify signals from trees with foliage damage caused by insect herbivores. They also showed that the predation rate by birds varied along an elevation gradient with the highest predation rate at a rather low elevation (700 m a.s.l.). The universal applicability of this result was highlighted by the study of Roslin et al. (2017). In a worldwide field experiment, artificial caterpillars were used to measure predation risk across different forest and shrubland habitats.

Overall, the experimental results supported the theory that predation (including both invertebrate and vertebrate predators) increases toward the equator (MacArthur 1969; Schemske et al. 2009), and also at lower elevations. Roslin et al. (2017) suggested that investigations using standardized comparisons of species interactions should be stimulated to reveal general patterns, e.g. geographical variations in predation pressure. Our findings in the present study add credence to the idea of using artificial caterpillars as a means to obtain standardized estimates of predation rates, at least in woodland habitats. The relationship between survival probabilities of caterpillars in relation to leaf damages might provide an opportunity to pinpoint context-dependent variations that could be used in applied science.

Management may affect bird predation pressure on canopy-living arthropods. But in the present study, variables that could be influenced by management (vegetation density, number bird individuals) were not included in the final model (Table 2). In a previous study, we found that clearance of understory may cause reduction in predation pressure on arthropod abundance (Heyman and Gunnarsson 2011). This was likely due to a decrease in bird abundance by, on average, 37% in cleared plots (Heyman 2010). But a review of experimental studies on management effects showed that clearance gave mixed results and suggested that bird communities’ responses were highly species specific (Heyman et al. 2017). A positive relationship between bird abundance and predation rate on caterpillars is theoretically expected and has been observed in other studies that employed the method of estimating bird predation rate using plasticine larvae (e.g. Barbaro et al. 2012, 2014; Bereczki et al. 2014; Sam et al. 2015). We could not find any significant influence of the abundance of avian insectivores on the relationship between caterpillar survival and leaf damage at our experimental sites. The reason might be that we included all insectivorous species in the point counts at our sites and this might be misleading. Studies in tropical agroforestry systems have indicated that a single species can be the main driver of predation on caterpillars (Maas et al. 2015). We have no data indicating which species attacked the caterpillars in our study sites. But the variation in beak and hack marks on the larvae suggested that multiple species of birds caused the injuries.

In conclusion, the evidence that avian insectivores can help to protect plants is well supported by multiple studies (e.g. Van Bael et al. 2008; Mooney et al. 2010; Mäntylä et al. 2011) and the results in the present study add further credence to this idea by showing a negative relationship between survival probability of caterpillars and leaf damage. Our findings on oak trees suggest that the survival probability of caterpillars decreases rapidly at 15–20% leaf damage. Sam et al. (2015) removed ca 5% leaf area in their experiment and recorded a significant effect on predation rate, which suggests that mechanically damaged leaves might attract more birds to manipulated trees. This could be a useful strategy to control insect herbivores. Manipulation of leaf damages could increase the yield in various crop systems through increasing predation pressure by avian insectivores on insects, especially in tropical agroforestry systems (Van Bael et al. 2008; Karp et al. 2013; Maas et al. 2013, 2015).

## Electronic supplementary material

Below is the link to the electronic supplementary material.

Supplementary material 1 (DOCX 14 kb)

Supplementary material 2 (DOCX 13 kb)

Supplementary material 3 (CSV 1 kb)
