# Supplementary material for: Predation by avian insectivores on caterpillars is linked to leaf damage on oak (Quercus robur)
Source: Oecologia. 2018 Aug 16;188(3):733–41. doi: 10.1007/s00442-018-4234-z (PMC6208694; doi:10.1007/s00442-018-4234-z)
Supplement: Supplementary file 1 — Supplementary material 1 (DOCX 14 kb) [file 442_2018_4234_MOESM1_ESM.docx]

Appendix 1.

##

# Code for

# 'Predation by avian insectivores on caterpillars is linked to leaf damages on oak

# (Quercus robur)'

# by Bengt Gunnarsson, Jonas Wallin, Jenny Klingberg

#

#

# the number bootstrap samples (nboot) is set to 20 for fast run, the figure in the article

# is done with 10K samples.

#

# Date: 2017-10-07

##

rm(list=ls())

graphics.off()

library(lme4)

library(arm)

set.seed(43)

save.fig = T

ntree_PI = 13 #median antalet

data.trad <- read.csv('../Data/caterpillarTree.csv', header=T, sep=" ")

data.trad$omrade_type <- data.trad$omrade -1

data.trad$omrade <- factor(data.trad$omrade)

#data.trad$betning <- data.trad$betning- mean(data.trad$betning)

n <- length(data.trad[,1])

data.trad$obs <- 1:n

nboot <- 10^4

n_grid <- 200

critval <- 2

cat('corr(betning,ccover) = ',round(cor(data.trad$betning,data.trad$ccover), 2),'\n')

###

#basic fit

form <- formula(cbind(y, n- y) ~ betning + (1|omrade))

model.trad = glmer( formula = form,

data = data.trad,

family = binomial(link = 'logit'),

control=glmerControl(

optimizer="bobyqa",

optCtrl=list(maxfun=5000000) ) )

###

sm <- summary(model.trad)

cat('beta_1 = ' , round(sm$coefficients[2,1],1),'\n')

cat('p.value = ', round(sm$coefficients[2,4],4),'\n')

conf_beta <- confint(model.trad,method='boot', nsim = nboot, level=0.95)

betning_grid <- seq(0,0.3,length=n_grid)

X <- cbind(1, betning_grid)

#bootstrap run

predFun<-function(.) invlogit( X%*%fixef(.) )

bb<-bootMer(model.trad,

FUN=predFun,

nsim=nboot)

boot_CI <- apply(bb$t,2,function(x) quantile(x, c(0.025,0.975)))

bb_Pred <- apply(bb$t,2, function(x) rbinom(n=length(x), ntree_PI,prob=x))/ntree_PI

boot_PI <- apply(bb_Pred,2,function(x) quantile(x, c(0.025,0.975)))

if(save.fig){

pdf('Fig5.pdf')

}else{

x11()

}

plot(data.trad$betning, data.trad$y/data.trad$n, pch = data.trad$omrade_type,xlab='Leaf damage', ylab = 'Survival probability')

lines(betning_grid, invlogit( cbind(1, betning_grid) %*% fixef(model.trad) ), col='blue',lwd=2)

lines(betning_grid, boot_CI[1, ], col='blue',lwd=2, lty=2)

lines(betning_grid, boot_CI[2, ], col='blue',lwd=2, lty=2)

lines(betning_grid, boot_PI[1, ], col='red',lwd=2, lty=3)

lines(betning_grid, boot_PI[2, ], col='red',lwd=2, lty=3)

if(save.fig)

dev.off()
