# Supplementary material for: Predation by avian insectivores on caterpillars is linked to leaf damage on oak (Quercus robur)
Source: Oecologia. 2018 Aug 16;188(3):733–41. doi: 10.1007/s00442-018-4234-z (PMC6208694; doi:10.1007/s00442-018-4234-z)
Supplement: Supplementary file 2 — Supplementary material 2 (DOCX 13 kb) [file 442_2018_4234_MOESM2_ESM.docx]

Appendix 2.

##

# AIC comparison with various models

#

# 'Predation by avian insectivores on caterpillars is linked to leaf damages on oak

# (Quercus robur)'

# by Bengt Gunnarsson, Jonas Wallin, Jenny Klingberg

#

##

rm(list=ls())

library(lme4)

set.seed(4)

data.trad <- read.csv('../Data/caterpillarTree.csv', header=T, sep=" ")

data.trad$omrade_type <- data.trad$omrade -1

data.trad$omrade <- factor(data.trad$omrade)

form <- formula(cbind(y, n- y) ~ betning + LAI + faglar + (1|omrade))

model.trad = glmer( formula = form,

data = data.trad,

family = binomial(link = 'logit'),

control=glmerControl(

optimizer="bobyqa",

optCtrl=list(maxfun=500000) ) )

print(drop1(model.trad))

#drop faglar

form <- formula(cbind(y, n- y) ~ betning + LAI + (1|omrade))

model.trad = glmer( formula = form,

data = data.trad,

family = binomial(link = 'logit'),

control=glmerControl(

optimizer="bobyqa",

optCtrl=list(maxfun=500000) ) )

print(drop1(model.trad))

#drop LAI

form <- formula(cbind(y, n- y) ~ betning + faglar + (1|omrade))

model.trad = glmer( formula = form,

data = data.trad,

family = binomial(link = 'logit'),

control=glmerControl(

optimizer="bobyqa",

optCtrl=list(maxfun=500000) ) )

print(drop1(model.trad))
